# Associations between retail food environment and the nutritional quality of food purchases in French households: The Mont’Panier cross-sectional study

**DOI:** 10.1371/journal.pone.0267639

**Published:** 2022-04-27

**Authors:** Daisy Recchia, Marlène Perignon, Pascaline Rollet, Simon Vonthron, Marion Tharrey, Nicole Darmon, Thierry Feuillet, Caroline Méjean, on behalf of the Surfood-Foodscapes working group

**Affiliations:** 1 MoISA, Univ Montpellier, CIRAD, CIHEAM-IAMM, INRAE, Institut Agro, IRD, Montpellier, France; 2 INNOVATION, Univ Montpellier, CIRAD, INRAE, Institut Agro, Montpellier, France; 3 University Paris 8, LADYSS, UMR 7533 CNRS, Saint-Denis, France; 4 Nutritional Epidemiology Research Team (EREN), Inserm, Inrae, Cnam, Epidemiology and Statistics Research Center, Bobigny, France; The University of Hong Kong, HONG KONG

## Abstract

**Purpose:**

The purpose of this study was to assess whether the retail food environment, measured by multiple indicators around the home and in activity space, was associated with the nutritional quality of food purchases.

**Methods:**

This cross-sectional study included 462 households from a quota sampling survey conducted in the south of France (Montpellier Metropolitan Area). The revised Healthy Purchase Index was implemented in order to assess the nutritional quality of food purchases. Food environment indicators (presence, number, relative density and proximity of food outlets) were calculated around the home and in activity space using a geographical information system. Six different types of food outlets were studied: supermarkets, markets, greengrocers, bakeries, other specialized food stores (butcher’s, fishmonger’s and dairy stores) and small grocery stores. Associations between food environment and the nutritional quality of food purchases were assessed using multilevel models, and geographically weighted regressions to account for spatial non-stationarity. Models were adjusted for households’ socioeconomic and demographic characteristics.

**Results:**

The nutritional quality of food purchases was positively associated with the number of greengrocers around the home (1 vs. 0: β = 0.25, 95%CI = [0.01, 0.49]; >1 vs. 0: β = 0.25, 95%CI = [0.00, 0.50]), but negatively associated with the number of markets around the home (1 vs. 0: β = -0.20, 95%CI = [-0.40, 0.00]; >1 vs. 0: β = -0.37, 95%CI = [-0.69, -0.06]). These associations varied across space in the area studied. For lower income households, the number of greengrocers in activity space was positively associated with the nutritional quality of food purchases (1 vs. 0: β = 0.70, 95%CI = [0.12, 1.3]; >1 vs. 0: β = 0.67, 95%CI = [0.22, 1.1]).

**Conclusions:**

Greengrocers might be an effective type of food store for promoting healthier dietary behaviors. Further studies, particularly interventional studies, are needed to confirm these results in order to guide public health policies in actions designed to improve the food environment.

## Introduction

Eating behaviors and dietary intake result from complex interactions between individual and contextual characteristics. Environmental features such as the food environment or “foodscape” [1]–defined as the physical, sociocultural and economic space in which people encounter meals and foods [2]–might be associated with dietary intake, physical activity and obesity [3]. Research on the role of the food environment in promoting or hindering healthy eating is continuously growing [4–9]. Dietary components, such as fruit and vegetable (FV) consumption, is by far the most common outcome used to assess eating behaviors [3]. Measuring overall diet quality using indices may, however, be more fitting as we do not consume separate food groups, but meals including a variety of food groups, where interactions between foods can occur [10].

Conclusions from previous literature reviews on the associations between food environment and overall diet quality draw mixed results [4–6]. To cite some examples, Bivoltsis et al. highlighted results of a Canadian study where no conclusive association between food environment and the Canadian adapted Healthy Eating Index (HEI-C) was found [11], as well as results from an Irish study where a significant negative association between distance to supermarket and adherence to the Dietary Approaches to Stop Hypertension (DASH) was reported [12]. Stevenson et al. focused on studies carried out in Canada, where mostly no association between food environment and diet quality was found [4]. Rahmanian et al. reviewed, among others, two US studies, where greater proximity to supermarkets was associated with better diet quality in pregnant women (DQI-P) [13] and where participants living in best-ranked food environments were more likely to have a higher Alternate Healthy Eating Index (AHEI) score [14].

The heterogeneity of these results might be due to methodological limitations [15]. The great variability of food environment indicators (presence, number, proximity, absolute density and relative density) and of types of food outlet, make it difficult to compare study results with each other [16]. In addition, the investigation of food environment exposure was often limited to the surrounding residential areas and neglected the influence of other non-residential places linked to social activities and travel behaviors [17].

The majority of works evaluating relationships between food environment and eating behaviors have been conducted in the United States [3]. Studies evaluating these relationships in France remain scarce [18–21], yet food shopping behaviors are deeply linked with consumers’ food culture, making analysis in different geographical settings all the more important [22, 23].

The Montpellier Metropolitan Area (MMA), an urban area in the south of France, presented itself as an interesting study field to investigate the impact of = food environment on dietary practices given the support of local politicians and interest in this specific issue. Accordingly, we aimed to examine whether retail food environment (RFE) was associated with the overall nutritional quality of food purchases in the MMA. In this study, the RFE was measured using multiple indicators (presence, number, relative density and proximity of six types of food outlet), and food purchases were assessed by objective purchasing data in French households. Beyond the scientific value of this work, which uses an original approach to assess RFE around the home and in activity space, our study might help guide cities looking to improve their RFE.

## Methods

### Study population

This study was carried out as a part of the Surfood-Foodscapes project (https://www.foodscapes.fr/en), which was designed to assess the impacts of urban foodscapes on peoples’ food styles in the MMA, the seventh-largest urban area in France. Analyses were conducted using data from the Mont’Panier cross-sectional study (https://www.etude-montpanier.com) and from the JArDinS study [24]. Participants of both studies were recruited based on the same call for participation, targeting volunteer households living in the MMA. Both studies were carried out from May 2018 to December 2019, using the same data collection methods. Households in both samples had similar socioeconomic and demographic characteristics, and the results of sensitivity analysis with or without JArDinS households were also very similar. We thus decided to keep the households from the JArDinS study in the final analysis sample in order to increase statistical power of analyses.

In order to be included in the study, participants had to be 18 years or older, live in the MMA and participate at least to some degree in grocery shopping. Using sociodemographic data on the MMA from the French National Institute of Statistics (*INSEE*), we performed quota sampling based on household composition (one adult, multiple adults, one adult with at least one child, and multiple adults with at least one child), and age of head of household (< 30 years, 30–50 years and > 50 years).

The Mont’Panier study and the JarDinS study were conducted in accordance with the guidelines laid down in the Declaration of Helsinki, and all procedures were approved by the Institutional Review Board of the French Institute for Health and Medical Research (IRB Inserm no. IRB00003888 IORG0003254 FWA00005831) and were registered to the *Commission Nationale Informatique et Libertés*. Written electronic informed consent to participate in the study was obtained after a thorough explanation of the study to each participant. Participants would receive a €15voucher when returning all duly completed data collection materials.

### Retail food environment assessment

In this study, we focused on six different types of food outlet: supermarkets, markets (indoor and outdoor markets), greengrocers, bakeries, other specialized food stores (butcher’s, fishmonger’s and dairy stores) and small grocery stores. The classification of food outlet type was carried out based on SIRENE’s food store classification. SIRENE is a French national business and establishment register database that is managed by the INSEE, recording the identity of all active companies and their establishments in France. In France, most meals are consumed at home, as highlighted in the Third French Individual and National Food Consumption report (INCA 3) [25], which is why we chose to focus on RFE for at home food consumption, excluding restaurants, fast-food outlets, etc. We excluded food purchased to be consumed away-from-home when calculating the nutritional quality of households’ food purchases for the same reason.

The location of food stores was obtained through the SIRENE database. The SIRENE database provides information on the French directory of active companies and establishments. This database was first cross-checked using OpenStreetMaps (OSM). OSM provides open data on companies and establishments that can be updated and enriched by external contributors. Online searches on Google Maps, company websites of the major food retailers, and city websites (e.g. with information on local markets) were then performed to further verify the database. Final verifications were carried out through field observations on around 5% of the area studied: the information provided on location and type of food outlet by the database was validated through ground-truthing in the city-region of Montpellier between May 2018 and January 2019 [26].

For this study, only household members who participated in grocery shopping (even if they participated only a little) would be considered for the geocoding step. The addresses of their current home and places of main activity (i.e. work and/or other places they reported visiting at least once a week) were collected through an online questionnaire. In addition, mode of transport (e.g. walking, cycling, driving) both to and from these addresses was also obtained for activity space calculations, i.e. to determine the buffer size of journeys (explained below). The addresses collected were then geocoded using QGIS v3.4.7. With the geocoded addresses of food outlets, homes and household members’ places of main activity, RFE was assessed in two exposure environments using geographical information systems. Firstly, RFE was assessed around homes, and secondly, RFE was assessed in the activity space of households, defined as the exposure environment including areas around the home, around household members’ places of main activity, and commuting routes between those places.

RFE around homes was estimated using four types of indicator: number, presence and proximity of each food outlet type, and relative density of food stores selling FV. In France, general food stores, namely supermarkets, small grocery stores and greengrocers sell FV and are thus considered as such in our study. The proximity of food stores was calculated by assessing the shortest road network distance between the nearest food outlet relative to each home address. The number of each type of food outlet was calculated within a 500-meter (or 1000-meter) road network distance around each home address. As a large proportion of households had no supermarket, market or greengrocer within 500 meters of the home, a larger and more relevant buffer of 1000 meters was used for these types of outlet [16]. The number of food outlets was used to calculate the presence (binary count) and relative density of food stores selling FV (number of food outlets selling FV across the total number of food outlets).

RFE in the activity space of households was estimated using three types of indicator: number and presence of each type of food outlet, and relative density of food stores selling FV. Proximity, being commonly related to a specific place, was not estimated in the activity space of households which is composed of areas around multiple places. The number of each food outlet type in the activity space of households was calculated within a 500-meter road network distance around the home and other places of activity, as well as 100 meters or 300 meters along commuting routes between those places. Commuting routes were modelled based on the shortest street network distance and a specific buffer was used depending on the modes of transport declared in the online questionnaire; we used a 100-meter buffer for walking and cycling journeys, and a 300-meter buffer for car journeys.

Given the non-normal distribution of RFE indicators, mainly because of the high amount of zeros, categorization of RFE variables was undertaken according to the distribution of each variable, and in such a manner that the interpretation of results would make sense. The number of food outlet variables were categorized into three groups: none, one, and more than one food outlet, except for bakeries, other specialized food stores and small grocery stores when considered in the activity space of households. These three variables were then categorized into three other groups (0–3; 4–10; >10), because distributions with none, one, and more than one categories were too uneven (e.g. no household with no or only one bakery in its activity space given the high amount of bakeries). The proximity of food outlets was also categorized into three groups: <500 meters; 500–1000 meters; >1000 meters.

### Nutritional quality of monthly food purchases

In order to assess the food supplies of households, participants were asked to complete a food supply diary, in which they had to list all foods and drinks purchased (including online purchases), harvested, or received for at-home consumption, over a one-month period. Step-by-step instructions on how to complete the food supply diary was given to participants and also specified at the front of the diary. For each food purchase made, participants were asked to collect grocery cash register receipts. When grocery cash register receipts were not available, participants were asked to provide details of foods purchased, received or harvested (name, quantity and expense incurred when applicable). Missing data concerning expenses for food items were imputed using the INSEE database and price were found on food store websites. Mean prices were found on food store websites. Mean prices per kg were multiplied by the quantity (kg) when quantity was available and, when quantity was missing, quantity was first imputed per mean quantity of the given food item. We included imputed received and harvested food expenses in the r-HPI calculation in order to consider all foods that were available for at-home consumption; in addition, such practices might interfere with the relationships between the food environment and food purchases, as they might constitute an alternative food supply source to the commercial food offer. All food items were then classified into 11 groups and 27 subgroups in order to assess the share of expenses of major food groups. The nutritional quality of households’ food purchases was calculated by implementing the revised-Heathy Purchase Index (r-HPI).

The HPI is an index, based on share of expenses for specific food groups and sub-groups, developed to evaluate the nutritional quality of households’ monthly food purchases [27]. The r-HPI is a revised version of the HPI index, which is obtained by summing a purchase diversity sub-score and a purchase quality sub-score. It ranges from a minimum score of -8 to a maximum score of 17 points, where a higher score reflects a higher quality of the household’s monthly food purchases. The diversity sub-score ranges from zero to five points and is composed of five food groups. Each food group contributes to the diversity sub-score with one added point if the score criterion is met (one point is attributed when the share of expenses for: fruits ≥ 2.8%, vegetables ≥ 3.5%, starches ≥ 2.3%, dairy ≥ 8.2%, and meat, eggs and fish ≥ 19.7%). The quality sub-score is calculated using ten food groups: fruits and vegetables, cheeses, milks and yogurts, eggs and poultries, fish, red meat, processed meat, fats, starches and discretionary foods. The detailed description of the scoring system is presented in S1 Table.

### Covariates

Socioeconomic and demographic characteristics were obtained from the online questionnaire. Analyses accounted for household composition (one adult, multiple adults, one adult with at least one child, and multiple adults with at least one child), income per unit of consumption (<€1110/month, €1110-2000/month, >€2000/month, and does not wish to respond), age group of heads of household (<30 years, 30–50 years and >50 years), and away-from-home food consumption. The away-from-home food consumption variable was calculated using the self-reported answers of each household member to four questions concerning the frequency of meals (per month) consumed away from home according to the type of place (canteen, restaurant, fast-food outlet and a relative’s/friend’s home). Days of away-from-home food consumption per household was calculated with the total number of meals per month reported as consumed away from home for each household member divided by the number of household members and divided by two (considering lunch and dinner as potential away-from-home meals of the day).

### Statistical analysis

Descriptive statistics were expressed as means (standard deviation) and percentages. In order to examine the associations between households’ RFE and the nutritional quality of households’ food purchases, multivariate multilevel regression models with a random intercept were performed. Multilevel modelling provides the advantage of accounting for the hierarchical structure of the data and spatial dependency that typically characterize observations within spatial units [28]. Municipality was used as a random effect in our multilevel models, except for households living in the city of Montpellier, where a smaller unit was chosen, namely sub-districts. Analyses were conducted on standardized r-HPI scores (z-scores) [mean = 0, standard deviation (SD) = 1] so as to enable different variable scales to be directly compared.

Only covariates and RFE indicators associated with r-HPI at a 0.2 significance level in bi-variate analyses (linear models or multilevel models) were retained for inclusion in the subsequent multivariate models. Interaction effects between income level and RFE indicators associated with r-HPI were tested and were included in multivariate models at a 0.1 significance level.

Separate multilevel regression analyses were performed for each type of RFE indicator, adjusted for socioeconomic and demographic household characteristics, away-from-home food consumption, and relevant significant interactions; seven sets of multivariate models were developed.

The first four models included the previously selected RFE indicators calculated around the home: a first model with indicators assessing the number of food store types, a second model with presence indicators, a third model with relative density indicators and a fourth model with proximity indicators.

The last three multivariate models were conducted with RFE indicators calculated in the activity space of households: a first model with number indicators, a second model with presence indicators, and a third model with relative density indicators. As stated above, we did not develop a model with proximity indicators in activity space, since proximity is commonly related to a specific place and not multiple places.

In this paper, we chose to focus on results with indicators of number over those of presence so as to avoid redundancy and because indicators of number enable us to assess a potential gradual association–taking into account a possible diversification of food offer and prices for the same type of food outlet. Only models with number, relative density and proximity indicators will be described, the results of models with presence indicators will, however, be available in supplementary materials.

Geographically weighted regression (GWR) models were performed in order to consider the possible spatial variations of the relationships between RFE around the home and the nutritional quality of food purchases (i.e. spatial non-stationarity). GWR was not performed for associations with RFE indicators calculated in the activity space of households, since local regression models are estimated using unique coordinate points (i.e. home addresses). Multilevel models and GWR are complementary statistical analysis methods. GWR enabled us to explore the associations studied on a finer spatial scale without the constraint of administrative boundaries, while multilevel models compensate for the limited statistical inferences of GWR models that lower their explanatory power [29]. GWR enables us to model spatially varying relationships by using a kernel function that calculates local regression models for each coordinate point (home address of the household) with more weight given to closer surrounding data points (other households’ home coordinates) [29]. In our analyses, we used a Gaussian kernel function with an adaptive bandwidth, number of nearest neighbors, as recommended when coordinate points are irregularly distributed across space [30], minimizing the corrected Akaike information criterion (AICc). The number of nearest neighbors was 460 for the GWR model with number indicators. The local β coefficients, as well as the corresponding t-values for the relationships studied, were mapped using R software.

Sensitivity analyses were performed on the 415 households of the Mont’Panier study, excluding the 47 participants of the JArDinS study. As the results of the sensitivity analyses, namely multivariate multilevel regressions, were very similar to those presented in this paper (i.e. significant results remained significant and the direction of associations remained the same), we did not include them in this paper.

All analyses were conducted using SAS, version 9.4 (SAS Institute, Cary, NC) and R (version 4.0.4). GWR models were performed using R package GWmodel [31]. The threshold for statistical significance was p < 0.05.

## Results

### Participant characteristics

Two sets of data were used for this paper, 415 households participating in the Mont’Panier survey and 47 households in the JArDinS study [24]. Analyses were conducted on households with complete data on RFE, food purchases and socioeconomic and demographic characteristics. After the exclusion of households with incomplete data, 462 households were included in the main analyses.

Households composed of several adults without children were the most represented household structure in our study sample, income per consumption unit levels were relatively well distributed among the study sample, and over one third of head of households were aged between 30 and 50 years (Table 1). The distribution of socioeconomic and demographic characteristics of households is relatively close to that of the population census of the study area (INSEE 2017) as shown in Table 1.

**Table 1:** Socioeconomic and demographic characteristics of households (N = 462).

	Mont’Panier (N = 462)	INSEE 2017 ^1^ (N = 254291)
	N (%)	%
**Household structure**		
One adult	150 (32.5%)	43.6%
One adult with at least one child	27 (5.8%)	10.1%
Multiple adults	176 (38.1%)	25.5%
Multiple adults with at least one child	109 (23.6%)	20.8%
**Income per unit of consumption**		
< €1110/month	130 (28.1%)	
€1110-2000/month	157 (34.0%)	
> €2000/month	147 (31.8%)	
Does not wish to respond	28 (6.1%)	
**Age of head of household**		
< 30 years	117 (25.3%)	21.4%
30–50 years	182 (39.4%)	33.1%
> 50 years	163 (35.3%)	45.5%

^1^ INSEE, Household Budget Survey 2017

The distribution of the sample according to the REF indicators is described in S2 Table. The number and proximity of supermarkets, markets, greengrocers, bakeries, other specialized food stores (butcher’s, fishmonger’s and dairy stores) and relative density of food stores selling FV are described around households’ homes and in their activity space (except for proximity indicators). The results for food outlet presence can be derived from the results for number of food outlets (categories 0 and 1 combined with >1). Most RFE indicators are well distributed among the three categories; however, some specificities can be noted. More than half of households live more than 1 km from a supermarket or market, but less than 500 m from a bakery. Also, more than half of households have no specialized food store within 500 m of their home, but most do have more than one supermarket or greengrocer in their activity space.

Concerning the nutritional quality of households’ food purchases, mean r-HPI was 9.2 (SD: 2.7), median r-HPI was 9.5, with a minimum score of minus two points and a maximum score of 15 points (r-HPI score ranges from a minimum score of -8 to a maximum score of 17 points).

### Nutritional quality of food purchases according to the socioeconomic characteristics of households

Household composition, income per unit of consumption, and head of household’s age group were significantly associated with the nutritional quality of food purchases. The results of bivariate linear models are presented in S3 Table. Households composed of multiple adults with no children had significantly better nutritional quality of food purchases compared to households composed of a single adult with no children, as did households with higher incomes compared to households with lower incomes (€1110-2000/month, >€2000/month vs. <€1110/month). Households with older heads of household (30–50 years, over 50 years vs., <30 years) also had a significantly better nutritional quality of food purchases.

### Retail food environment around homes

Adjusted associations between the number of food stores around the home and the nutritional quality of food purchases showed that the number of markets was significantly and inversely associated with the nutritional quality of food purchases, whereas the number of greengrocers was positively associated with the nutritional quality of food purchases (Table 2). These associations were adjusted on household structure, income per unit of consumption, age of head of household, and away-from-home food consumption. Households with a higher income level (> €2000/month vs. <€1110/month) seem to have higher a nutritional quality of food purchases, as do households whose heads of household are older (> 50 years vs. <30 years).

**Table 2:** Adjusted multilevel model of the associations between number of food outlets around the home and the nutritional quality of food purchases (r-HPI z-score) (N = 462).

	β	95% CI^1^	p-value
**Number of markets**			**0.033**
0			
1	**-0.20**	**-0.40, 0.00**	**0.048**
>1	**-0.37**	**-0.69, -0.06**	**0.021**
**Number of greengrocers**			0.069
0			
1	**0.25**	**0.01, 0.49**	**0.045**
>1	**0.25**	**0.00, 0.50**	**0.049**
**Number of bakeries**			0.5
0			
1	-0.10	-0.33, 0.13	0.4
>1	0.02	-0.24, 0.28	0.9
**Number of small grocery stores**			0.5
0			
1	-0.16	-0.42, 0.10	0.2
>1	-0.05	-0.32, 0.21	0.7
**Household structure**			0.4
One adult			
One adult with at least one child	0.04	-0.37, 0.46	0.8
Multiple adults	0.19	-0.02, 0.41	0.080
Multiple adults with at least one child	0.08	-0.19, 0.35	0.6
**Income per unit of consumption**			0.081
< €1110/month			
€1110-2000/month	0.23	-0.01, 0.46	0.060
> €2000/month	**0.30**	**0.04, 0.55**	**0.023**
Does not wish to respond	0.40	-0.01, 0.81	0.058
**Age of head of household**			**<0.001**
< 30 years			
30–50 years	0.17	-0.13, 0.46	0.3
> 50 years	**0.51**	**0.21, 0.81**	**<0.001**
**Away-from-home food consumption**	-0.01	-0.02, 0.00	0.068

^1^CI = Confidence Interval

Number of supermarkets and other specialized stores around the home were not included in this multivariate model because they had p-values > 0.2 in bivariate analyses.

**Fig 1:**
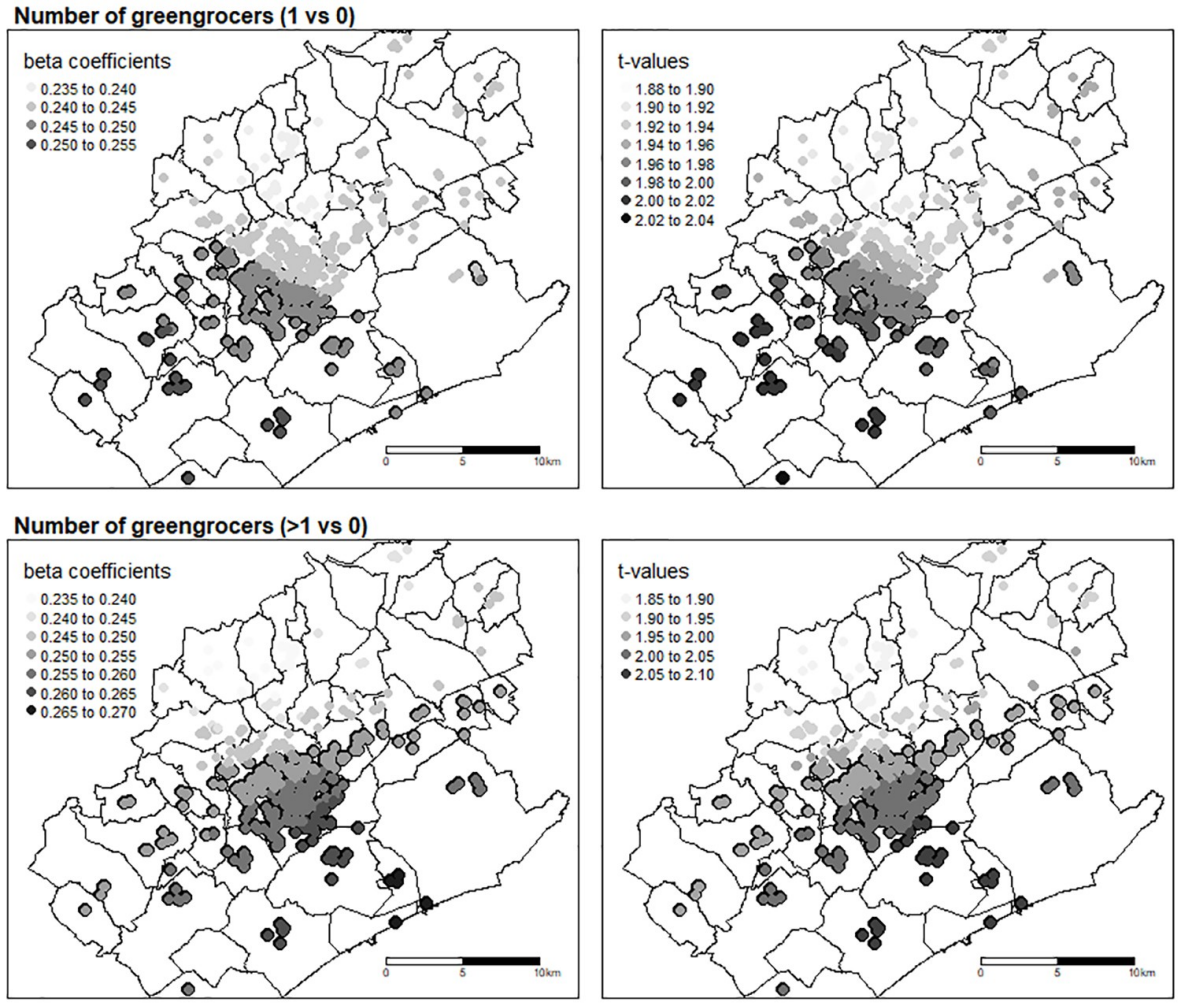
Spatial variations of β coefficients and t-values derived from a GWR model assessing the adjusted associations between the number of greengrocers around the home and the nutritional quality of food purchases (r-HPI z-score) (N = 462). Adjustments were made on household composition, income per unit of consumption, head of household’s age group, away-from-home food consumption, and number of markets, bakeries and small grocery stores. Source for borders: IGN 2017 BD TOPO^**®**^.

The results of GWR for the associations of nutritional quality of households’ food purchases with number of greengrocers are mapped in Fig 1. Only maps for number of greengrocers are presented in this paper, since greengrocers were the main type of food outlet that stood out in our study with persistent and robust significant associations. Spatial variation with a south-north gradient can be seen on these maps, with higher β coefficients (1 vs. 0: 0.245 to 0.255; >1 vs. 0: 0.250 to 0.270) in the south of the area studied and lower coefficients (1 vs. 0: 0.235 to 0.245; >1 vs. 0: 0.235 to 0.250) in the northern part. Associations are significant in the south of the study area (significance being represented by black circles), but not significant in the north of the MMA (t-value <1.96).

The results of the adjusted associations between the presence of food stores around the home and the nutritional quality of food purchases are shown in S4 Table. The presence of only two types of food outlet around the home was significantly associated with the nutritional quality of food purchases: this association was positive for the presence of greengrocers and negative for the presence of markets. These results, which are coherent with the results obtained for number of food outlet indicators, show that the mere presence of these two types of food store is sufficient to observe significant associations.

Bivariate association analysis between the relative density of food stores selling FV around households’ homes and the nutritional quality of food purchases was not statistically significant (0.4–0.5 vs. <0.4: β = -0.11, 95%CI = [-0.34, 0.12]; >0.6 vs. <0.4: β = 0.09, 95%CI = [-0.13, 0.31]; global p-value: 0.3).

Adjusted associations between the proximity of food store types around households’ homes and the nutritional quality of food purchases were also not significant (Table 3). Only associations with covariates turned out to be significant; higher income households and households with older heads of household having a higher r-HPI score and households consuming more meals away from home having a lower r-HPI score.

**Table 3:** Adjusted multilevel model of the associations between proximity of food outlets around the home and the nutritional quality of food purchases (r-HPI z-score) (N = 462).

	β	95% CI^1^	p-value
**Proximity of bakeries**			0.8
<500 m			
500–1000 m	0.06	-0.15, 0.28	0.6
>1000 m	0.07	-0.23, 0.38	0.6
**Proximity of small grocery stores**			0.6
<500 m			
500–1000 m	0.10	-0.13, 0.33	0.4
>1000 m	0.01	-0.22, 0.24	>0.9
**Household structure**			0.3
One adult			
One adult with at least one child	-0.01	-0.42, 0.40	>0.9
Multiple adults	0.20	-0.02, 0.41	0.073
Multiple adults with at least one child	0.11	-0.16, 0.38	0.4
**Income per unit of consumption**			0.075
< €1110/month			
€1110-2000/month	0.24	0.00, 0.48	0.050
> €2000/month	**0.30**	**0.05, 0.56**	**0.020**
Does not wish to respond	0.40	-0.02, 0.81	0.062
**Age of head of household**			**<0.001**
< 30 years			
30–50 years	0.22	-0.07, 0.52	0.14
> 50 years	**0.56**	**0.26, 0.85**	**<0.001**
**Away-from-home food consumption**	**-0.01**	**-0.02, 0.00**	**0.046**

^1^CI = Confidence Interval

The proximity of supermarkets, markets, greengrocers and other specialized stores around the home was not included in this multivariate model because it had p-values >0.2 in bivariate analyses.

### Retail food environment in the activity space of households

Analyses of associations between the number of food stores in the activity space of households and the nutritional quality of food purchases were stratified for income level, because an interaction between the number of greengrocers and households’ income levels was found (global p-value = 0.011). Associations showed that the number of greengrocers in the activity space of households was significantly and positively associated with the nutritional quality of food purchases in households with lower incomes (Table 4). Results of the associations between the number of food outlets in activity space and the nutritional quality of food purchases without income stratification are presented in S5 Table.

**Table 4:** Adjusted multilevel model (stratified by income level) of the associations between number of food outlets and activity space and nutritional quality of food purchases (r-HPI z-score) (N = 462).

	< €1110/month (N = 130)			€1110-2000/month (N = 157)			> €2000/month (N = 147)			Does not wish to respond (N = 28)		
	β	95% CI^1^	p	β	95% CI^1^	p	β	95% CI^1^	p	β	95% CI^1^	p
**Number of greengrocers**			**0.006**			0.5			0.5			**<0.001**
0												
1	**0.70**	**0.12, 1.3**	**0.020**	0.00	-0.48, 0.49	>0.9	0.26	-0.25, 0.77	0.3	**0.87**	**0.06, 1.7**	**0.037**
>1	**0.67**	**0.22, 1.1**	**0.004**	-0.18	-0.53, 0.17	0.3	0.03	-0.39, 0.46	0.9	**1.1**	**0.44, 1.7**	**0.003**
**Household structure**			0.12			0.5			>0.9			0.3
One adult												
One adult with at least one child	-0.14	-0.93, 0.64	0.7	0.09	-0.49, 0.67	0.8	-0.05	-1.4, 1.3	>0.9	0.65	-1.0, 2.3	0.4
Multiple adults	**0.48**	**0.01, 0.94**	**0.044**	0.02	-0.32, 0.37	0.9	0.07	-0.32, 0.47	0.7	-0.05	-0.69, 0.58	0.9
Multiple adults with at least one child	-0.10	-0.74, 0.55	0.8	0.31	-0.10, 0.71	0.13	0.08	-0.38, 0.54	0.7	0.77	-0.65, 2.2	0.2
**Age of head of household**			0.073			0.6			**<0.001**			**0.002**
< 30 years												
30–50 years	0.47	-0.10, 1.1	0.11	-0.06	-0.56, 0.44	0.8	-0.01	-0.73, 0.71	>0.9	**2.8**	**1.0, 4.7**	**0.005**
> 50 years	**0.58**	**0.07, 1.1**	**0.027**	0.11	-0.41, 0.62	0.7	0.70	-0.04, 1.4	0.063	**2.0**	**0.25, 3.8**	**0.028**
**Away-from-home food consumption**	0.00	-0.02, 0.03	0.8	-0.02	-0.04, 0.00	0.055	-0.01	-0.03, 0.01	0.3	**-0.05**	**-0.09,-0.01**	**0.011**

^1^CI = Confidence Interval

The number of supermarkets, markets, bakeries, other specialized stores and small grocery stores in activity space was not included in this multivariate model because it had p-values >0.2 in bivariate analyses. The activity space includes areas around the home, around household members’ places of main activity, and commuting routes between those places.

The results of adjusted associations between the presence of food stores in the activity space of households and the nutritional quality of food purchases are presented in S6 Table. Analyses were stratified for income level because an interaction between the presence of greengrocers and households’ income level was found (global p-value <0.001). Associations showed that even the mere presence of greengrocers in the activity space of households was significantly and positively associated with the nutritional quality of food purchases in households with lower incomes. The results of the associations between presence of food outlets in activity space and the nutritional quality of food purchases without income stratification are presented in S7 Table. Once again, the results show that the presence of greengrocers in the activity space of households was significantly and positively associated with the nutritional quality of food purchases.

Bivariate association analysis between the relative density of food stores selling FV in the activity space of households and the nutritional quality of food purchases was not significant (0.4–0.5 vs. <0.4: β = -0.10, 95%CI = [-0.38, 0.18]; >0.6 vs. <0.4: β = -0.01, 95%CI = [-0.31, 0.31]; global p-value: 0.6).

## Discussion

To the best of our knowledge, this is the first study that explores the relationships between food environment and dietary behaviors by assessing the overall nutritional quality of households’ food purchases, and two scales of the RFE (around households’ homes and in their activity space). This study found that having greengrocers around the home and in activity space was associated with higher nutritional quality of food purchases; this association varied according to income level for greengrocers in activity space. Besides, the number of markets around households’ homes was associated with lower nutritional quality of food purchases, while no significant association was found for other types of food outlet.

There was no association between the proximity of food stores around households’ homes and nutritional quality of households’ food purchases. Nor did we find any associations between the relative density of food stores selling FV and the nutritional quality of food purchases. Only indicators of number and presence of food outlets showed significant associations with the nutritional quality of households’ food purchases. Types of spatial exposure measurement were compared in a recent systematic review [5], revealing that availability measures (number, presence) may return more robust and significant associations with dietary outcomes than accessibility measures (proximity), as is the case in our study. Choice and concentration of food outlets may have a greater influence on diet than distance to the closest food outlet [5].

Regarding household socioeconomic and demographic characteristics, households composed of multiple adults, with older heads of household or higher incomes had food purchases of better nutritional quality (results of bivariate linear models); similar results have been found in other studies [32, 33].

As in ours, another study reported significant associations between greater access to greengrocers and better dietary quality [34]. Likewise, greengrocers have previously been associated with FV purchasing [35, 36]. Other papers, however, did not find any significant association between availability or accessibility of greengrocers and FV consumption [37, 38]. Results on the associations between greengrocers and diet or purchasing habits are thus mixed. In our study, this association was positive (better nutritional quality of food purchases), which is coherent with following adjusted means of nutritional quality of food purchases according to greengrocer frequentation status (yes N = 121: mean = 9.55, SD = 0.24; no N = 341: mean = 9.09, SD = 0.14; p = 0.01). The availability of greengrocers might broaden and diversify the local food outlet offer, and consequently ease physical access to FV [35, 36, 39]. In a hypercompetitive food environment, (i.e. where food outlets are numerous and diversified), it is especially important to give the consumer a chance to choose food stores that encourage healthy, enjoyable and sustainable eating. In France, greengrocers engage in the distribution of fresh FV, from wholesalers or local producers. French households, concerned about the quality of food products, might prefer to buy FV from greengrocers, which tend to be seen as fresher and more appealing than the FV available in supermarkets. Even though supermarkets still are the main source of food supply in France [40], southern French households are still regularly shopping in smaller specialist shops such as greengrocers, which are, for instance, more abundant in the French RFE compared to central England’s RFE [41]. Greengrocers, among other food shops, may favor responsible and sustainable consumption [42], making them an interesting choice for eco-friendly consumers.

In our study, GWR models, adjusted on socioeconomic and demographic characteristics and away-from-home food consumption, revealed that locally estimated coefficients varied across space with a south-north gradient in the MMA. The Olympic Regeneration in East London Study also found nonstationary relationships between food environment and dietary behavior (FV intake) [43]. This spatial heterogeneity might have an effect on consumers’ food choices. Consumers living in the south of the MMA seem to be more impacted by the association between the number of greengrocers around the home and the nutritional quality of food purchases. This spatial heterogeneity is interesting to note and should be considered for the implementation of actions locally designed to improve the food environment. Discussions with city council should specifically involve greengrocers and consider specificities of the south of the MMA.

Furthermore, multilevel analyses, stratified by income level, revealed that among low-income households, those exposed to a greengrocer in their activity space made monthly food purchases of better nutritional quality. These findings are consistent with previous research stating that low-income populations are more easily influenced by their surroundings than high-income populations [44–46].

While we did not find any other studies reporting associations between the availability of markets and the nutritional quality of food purchases, we did find studies reporting positive associations between access to mobile food markets and FV intake [47, 48]. However, our findings showed that having multiple markets around the home was associated with lower nutritional quality of monthly food purchases. These findings need to be considered with caution, since in our study, households that had frequented a market (at least once in the month studied) made (vs. did not make) food purchases of higher nutritional quality (yes N = 172: mean = 9.59, SD = 0.20; no N = 290: mean = 8.99, SD = 0.15; p<0.001). Unmeasured confounding factors may explain this unexpected inverse relationship. A study, aiming to understand bias in relationships between the food environment and diet quality, also stated that ignoring residual confounding may generate biased estimated effects of food outlets on diet [49]. In our study sample, households who had more than one market around their home were mostly households living in the city center, where the competition between food store types is greater. Open air-markets have a consistent disadvantage when competing with other types of food outlet, since they do not have a permanent structure and are thus only to be seen on specific days of the week, during specific hours, limiting markets’ influence on consumers’ purchasing behavior. A study assessing the potential contribution of markets to an urban food environment in terms of accessibility, produce variety, quality, and price, revealed that markets were open substantially fewer months, days, and hours, offered fewer fresh produce items, and were, on average, more expensive than other nearby stores offering fresh produce [50]. Depending on the markets, the food offer can substantially vary across and within countries, ranging from exclusively or mainly FV, to a large range of products including foods high in fat, sugar and salt (e.g. processed meats, cheeses, jams, pies and ready meals) [51, 52]. In addition, a recent study highlighted greengrocers as the most accessible FV outlet, compared to farmers’ markets which had lower availability and/or fewer varieties of FV [36].

With today’s competitive food environment contributing to rising obesity rates and, thus, to the increasing risk of chronic diseases, it is crucial for public health policies to establish food environments that promote healthy food choices, which consequently could turn out to be a substantial public health payoff in the future. Studies like this could help guide public health policies, facilitating the decision-making process to improve the food environment. Even though we did not find any statistically significant association between supermarkets and the nutritional quality of food purchases in our study, big food supply chains remain the main food supply source even in France [40], and are thus still to be considered for future interventions aiming to increase healthy food purchasing. Our results with supermarkets might have been non-significant, because the sole presence of supermarkets may not be sufficient to have an impact on consumer behavior; marketing strategies defined as the 4Ps (product, price, placement, promotion) used by supermarkets should be considered for interventions designed to increase healthy food purchasing, in particular the promotion of healthy food products [53]. Future interventions should also take into account the sustainability of food purchases and, given the COVID-19 context, it would also be wise to place a special focus on online food sales [53].

The strengths of our study include the use of objective data on dietary behavior (households’ monthly food purchases) based on the collection of households’ receipts over a one-month period. This allowed us to reduce the known declaration biases [54], often found with self-reported measures, such as food frequency questionnaires, 24-hour recalls, food records, or diaries and screeners. In addition, we chose to measure the overall nutritional quality of food purchases using a validated index, based on expenditure shares, which better reflects dietary behaviors compared to simple dietary components, such as FV consumption. Another strength is the combination of two statistical analysis methods, multilevel modelling and GWR, which are complementary methods [55]. GWR allowed us to take into account the non-stationarity of the associations studied, and to explore them at a finer spatial scale without the constraint of administrative boundaries, while multilevel models compensate for the limited statistical inferences of GWR models that lower their explanatory power. Furthermore, we used an original approach to measure households’ exposure to RFE, by taking into account household members’ main places of activity and their commuting journeys, in order to calculate the activity space of households. The importance of considering food environment not only around the home, but also in activity space has been highlighted in previous studies [17]. Moreover, as it has been stated in previous systematic reviews [15], the use of multiple RFE indicators (presence, number, proximity and relative density) in the assessment of relationships between food environment and dietary behavior is necessary to allow comparison with results of other studies. Finally, we studied six different types of food outlet, hence taking into account the diversity of food supply sources to which French households are exposed to, rather than limiting the analyses to usually studied types of food store(supermarkets and fast-food restaurants), which do not serve as the only sources of foods and drinks [56]. This point is especially important in a French urban setting, where specialized food stores such as greengrocers, bakeries and butchers, etc. seem to be more abundant and more frequently used than in other countries (e.g. central England) [41].

This study also has limitations. Firstly, caution is needed regarding extrapolation of these results to the entire French population as this study was limited to a metropolitan area located in the South of France. Secondly, the study’s cross-sectional design does not allow for drawing any conclusions on the causal relationships between food environment and the nutritional quality of food purchases. In addition, neighborhood self-selection (i.e. people selecting neighborhoods to live in which have the facilities and resources to suit their preferred lifestyle) was not taken into account in this study, which can lead to biased estimates of the associations between the food environment and eating behaviors [57] and brings up the potentiality of reverse causality of the associations studied. Finally, selection bias may also be an issue in this study, since households that volunteered to participate in a study on dietary behaviors may initially be more interested in nutrition and thus be more inclined to make healthier food purchases. However, in order to limit selection bias, quota sampling was performed based on household composition and age of head of household. Moreover, purchases might not always reflect consumption [58], which could be seen as another limit of our study; however, our study did not attempt to use food purchases as a proxy for food consumption. Indeed, our aim was to assess the relationships between food environment and the nutritional quality of food purchases, not diet quality. It may, nonetheless, prove to be a limit for comparison with other studies, which are using individual food consumption data and not household food purchasing data, as we did. In addition, the affordability or price of food products was not taken into account in our study, even though this might have an influence on the associations between RFE and the nutritional quality of households’ food purchases. Indeed, the food choices of consumers can be impacted by food prices, which might differ according to food item, but also according to food supply source and, even more so, when it concerns food consumed away from home (restaurants, fast-foods outlets, etc.). Be that as it may, purchases of foods that were consumed away from home were not included in estimation of the nutritional quality of food purchases. This might also be seen as a limit of our study, since away-from-home food consumption might be part of the untapped components for the nutritional quality of food purchases. In order to account for this bias, we made adjustments on away-from-home food consumption in our multivariate analyses.

## Conclusions

This paper presents an original approach when studying the relationships between food purchasing behaviors and food environment exposure assessed in activity space, taking into account exposure around the home, other places of activity (e.g. work place) and along commuting routes. Our study showed that households exposed to greengrocers make monthly food purchases of better nutritional quality, especially in low-income households. Only RFE indicators of number and presence of food outlets showed significant associations with the nutritional quality of households’ food purchases, suggesting that type and concentration of food outlet may have a greater influence on diet than distance to the closest food outlet.

Future studies should consider using multiple RFE indicators and take into account diverse types of food supply source in order to allow for study comparison. In addition, exposure to the food environment should be considered in activity space and not solely around the home. Furthermore, we recommend future studies in order to assess how RFE influences food purchases for at-home as well as away-from-home food consumption. Finally, natural experiment studies designed to explore causality should be conducted in order to assess the suggested effect of greengrocers on dietary behaviors. Consecutively, such research may help guide public health policies to implement actions designed to improve RFE, including actions that might contribute to decreased social inequalities with regard to diet.

## Supporting information

S1 Table Components and cut-offs used for calculation of the r-HPI. ^a^ Except where specified; ^b^ Fresh fruits, canned fruits, stewed fruits. ^c^ Fresh vegetables, vegetable soups, canned vegetables. ^d^ Potatoes, legumes, wholegrain products, bread rolls, fresh bread, pasta, rice, flour. ^e^ Milks & yoghurts, cheeses. ^f^ Red meat, processed meat, eggs & poultry, fish. ^g^ Vegetables, fruits, dried fruits & nuts. ^h^ Hard cheese, soft cheese, cream cheese. ^i^ Refrigerated and long-life milk, plain yoghurt, sweetened yoghurt, fruit yoghurt, yoghurt drink. ^j^ Hard-boiled egg, fried egg, omelet, chicken, duck, turkey. ^k^ Fresh fish, canned fish, shellfish, surimi. ^l^ Beef, pork, lamb. ^m^ Cured and cooked ham, sausages, bacon, pâté. ^n^ Vegetable fats: vegetable oil, margarine, salad dressing; animal fats: cream, butter. ^o^ Savory snacks, sugar sweetened beverages, calorie free beverages, fruit juices, sugared cereals, dairy desserts, sweet snacks, sauces. TF: Total Fats.
(DOCX)

S2 Table Characteristics of households’ retail food environment (N = 462). The results for presence of food outlets can be derived from the results for number of food outlets (categories 0 and 1 combined with >1). ^a^ Other specialized food stores include butcher’s, fishmonger’s and dairy stores. ^b^ The proximity of food stores was calculated by assessing the shortest road network distance between the nearest food outlets relative to each home address. ^c^ The number of food stores was measures within a 1000-meter road network distance around the home for supermarkets, markets and greengrocers and within a 500-meter road network distance around the home for bakeries, other specialized food stores and small grocery stores. ^d^ The number of food stores in activity space was calculated within a 500-meter road network distance around the home and other places of activity, as well as 100 meters or 300 meters along commuting routes between those places. A 100-meter buffer was used for walking and cycling journeys while a 300-meter buffer was used for car journeys. ^e^ Relative density of food stores selling fruits and vegetables was measured within a 500-meter road network distance around the home and calculated by dividing the number of food stores selling fruits and vegetables by the total number of food stores.
(DOCX)

S3 Table Bi-variate linear models of the associations between the socioeconomic and demographic characteristics of households and the nutritional quality of food purchases (r-HPI z-score) (N = 462). ^a^ CI = Confidence Interval.
(DOCX)

S4 Table Adjusted multilevel model of the associations between the presence of food outlets around the home and the nutritional quality of food purchases (r-HPI z-score) (N = 462). ^a^ CI = Confidence Interval; the presence of supermarkets and other specialized food stores around the home was not included in this multivariate model because it had p-values >0.2 in bivariate analyses.
(DOCX)

S5 Table Adjusted multilevel model of the associations between the number of food outlets in activity space and the nutritional quality of food purchases (r-HPI z-score) (N = 462). ^a^ CI = Confidence Interval; the number of supermarkets, markets, bakeries, other specialized stores and small grocery stores in activity space was not included in this multivariate model because it had p-values >0.2 in bivariate analyses. The activity space includes areas around the home, around household members’ places of main activity, and commuting routes between those places.
(DOCX)

S6 Table Adjusted multilevel model (stratified by income level) of the associations between the presence of food outlets in activity space and the nutritional quality of food purchases (r-HPI z-score) (N = 462). ^a^ CI = Confidence Interval; the presence of markets, bakeries, other specialized stores (butcher’s, fishmonger’s and dairy stores) and small grocery stores in activity space was not included in this multivariate model because it had p-values >0.2 in bivariate analyses. The activity space includes areas around the home, around household members’ places of main activity and commuting routes between those places.
(DOCX)

S7 Table Adjusted multilevel model of the associations between the presence of food outlets in activity space and the nutritional quality of food purchases (r-HPI z-score) (N = 462). ^a^ CI = Confidence Interval; the presence of markets, bakeries, other specialized stores (butcher’s, fishmonger’s and dairy stores) and small grocery stores in activity space was not included in this multivariate model because it had p-values >0.2 in bivariate analyses. The activity space includes areas around the home, around household members’ places of main activity and commuting routes between those places.
(DOCX)
