# Supplementary material for: Associations between retail food environment and the nutritional quality of food purchases in French households: The Mont’Panier cross-sectional study
Source: PLoS One. 2022 Apr 27;17(4):e0267639. doi: 10.1371/journal.pone.0267639 (PMC9045620; doi:10.1371/journal.pone.0267639)
Supplement: S1 Table — a Except where specified; b Fresh fruits, canned fruits, stewed fruits. c Fresh vegetables, vegetable soups, canned vegetables. d Potatoes, legumes, wholegrain products, bread rolls, fresh bread, pasta, rice, flour. e Milks & yoghurts, cheeses. f Red meat, processed meat, eggs & poultry, fish. g Vegetables, fruits, dried fruits & nuts. h Hard cheese, soft cheese, cream cheese. i Refrigerated and long-life milk, plain yoghurt, sweetened yoghurt, fruit yoghurt, yoghurt drink. j Hard-boiled egg, fried egg, omelet, chicken, duck, turkey. k Fresh fish, canned fish, shellfish, surimi. l Beef, pork, lamb. m Cured and cooked ham, sausages, bacon, pâté. n Vegetable fats: vegetable oil, margarine, salad dressing; animal fats: cream, butter. o Savory snacks, sugar sweetened beverages, calorie free beverages, fruit juices, sugared cereals, dairy desserts, sweet snacks, sauces. TF: Total Fats. (DOCX) [file pone.0267639.s001.docx]

| **DIVERSITY SUB-SCORE (0-5 points)** | | |
| --- | --- | --- |
| **Component** | **Cut-off (% of total food expenditure ^a^)** | **Score** |
| **Fruits ^b^** | [0-2,8[ | 0 |
|  | ≥ 2,8 | 1 |
| **Vegetables ^c^** | [0-3,5[ | 0 |
|  | ≥ 3,5 | 1 |
| **Starches ^d^** | [0-2,3[ | 0 |
|  | ≥ 2,3 | 1 |
| **Dairy products ^e^** | [0-8,2[ | 0 |
|  | ≥ 8,2 | 1 |
| **Meat/fish/eggs ^f^** | [0-19,7[ | 0 |
|  | ≥ 19,7 | 1 |
|  |  | |
| **QUALITY SUB-SCORE (-8 to +12 points)** | | |
| **Component** | **Cut-off (% of total food expenditure ^a^)** | **Score** |
| **Fruits & vegetables ^g^** | [0-6[ | 0 |
|  | [6-9[ | 1 |
|  | [9-12[ | 2 |
|  | [12-16[ | 3 |
|  | ≥ 16 | 4 |
| **Cheese ^h^** | < 4 | 1 |
|  | [4 - 8[ | 0 |
|  | ≥ 8 | -1 |
| **Milk & yogurts ^i^** | < 2 | 0.5 |
|  | [2 - 9[ | 1 |
|  | ≥ 9 | 0 |
| **Eggs & poultry ^j^** | < 3 | 0 |
|  | ≥ 3 | 1 |
| **Fish ^k^** | < 1,5 | 0 |
|  | [1,5 - 4[ | 1 |
|  | [4 - 7[ | 1.5 |
|  | ≥ 7 | 2 |
| **Red meat ^l^** | ≤ 21 | 0 |
|  | > 21 | -1 |
| **Processed meat ^m^** | ≤ 6 | 0 |
|  | ]6 - 10[ | -1 |
|  | ≥ 10 | -2 |
| **Fats ^n^** | TF = 0 | 0 |
|  | TF > 0 and animal fats [0 – 1] | 1 |
|  | Animal fats ]1 – 2] | 0 |
|  | Animal fats > 2 | -1 |
| **Starches ^d^** | Total starches = 0 | 0 |
|  | Total starches > 0 and unrefined starches = 0 | 0 |
|  | Unrefined starches ]0 - 18%[ of total starches | 1 |
|  | Unrefined starches [18 - 30%[ of total starches | 1.5 |
|  | Unrefined starches ≥ 30% of total starches | 2 |
| **Discretionary foods ^o^** | < 7 | 0 |
|  | [7 - 13[ | -1 |
|  | [13 - 18[ | -2 |
|  | ≥ 18 | -3 |
