# Supplementary material for: Associations between retail food environment and the nutritional quality of food purchases in French households: The Mont’Panier cross-sectional study
Source: PLoS One. 2022 Apr 27;17(4):e0267639. doi: 10.1371/journal.pone.0267639 (PMC9045620; doi:10.1371/journal.pone.0267639)
Supplement: S2 Table — The results for presence of food outlets can be derived from the results for number of food outlets (categories 0 and 1 combined with >1). a Other specialized food stores include butcher’s, fishmonger’s and dairy stores. b The proximity of food stores was calculated by assessing the shortest road network distance between the nearest food outlets relative to each home address. c The number of food stores was measures within a 1000-meter road network distance around the home for supermarkets, markets and greengrocers and within a 500-meter road network distance around the home for bakeries, other specialized food stores and small grocery stores. d The number of food stores in activity space was calculated within a 500-meter road network distance around the home and other places of activity, as well as 100 meters or 300 meters along commuting routes between those places. A 100-meter buffer was used for walking and cycling journeys while a 300-meter buffer was used for car journeys. e Relative density of food stores selling fruits and vegetables was measured within a 500-meter road network distance around the home and calculated by dividing the number of food stores selling fruits and vegetables by the total number of food stores. (DOCX) [file pone.0267639.s002.docx]

|  | **Food environment  around home** | | | | **Food environment  in activity space ^d^** | |
| --- | --- | --- | --- | --- | --- | --- |
|  | **Proximity ^b^** | | **Number ^c^** | | **Number** | |
|  | **Category** | **N (%)** | **Category** | **N (%)** | **Category** | **N (%)** |
| **Supermarkets** |  |  |  |  |  |  |
|  | <500m | 79 (17.1%) | 0 | 170 (36.8%) | 0 | 104 (22.5%) |
|  | 500-1000m | 148 (32.0%) | 1 | 144 (31.2%) | 1 | 90 (19.5%) |
|  | >1000m | 235 (50.9%) | >1 | 148 (32.0%) | >1 | 268 (58.0%) |
| **Markets** |  |  |  |  |  |  |
|  | <500m | 86 (18.6%) | 0 | 224 (48.5%) | 0 | 160 (34.6%) |
|  | 500-1000m | 124 (26.8%) | 1 | 156 (33.8%) | 1 | 127 (27.5%) |
|  | >1000m | 252 (54.5%) | >1 | 82 (17.7%) | >1 | 175 (37.9%) |
| **Greengrocers** |  |  |  |  |  |  |
|  | <500m | 122 (26.4%) | 0 | 153 (33.1%) | 0 | 103 (22.3%) |
|  | 500-1000m | 152 (32.9%) | 1 | 107 (23.2%) | 1 | 72 (15.6%) |
|  | >1000m | 188 (40.7%) | >1 | 202 (43.7%) | >1 | 287 (62.1%) |
| **Bakeries** |  |  |  |  |  |  |
|  | <500m | 264 (57.1%) | 0 | 161 (34.8%) | 0-3 | 146 (31.6%) |
|  | 500-1000m | 144 (31.2%) | 1 | 128 (27.7%) | 4-10 | 132 (28.6%) |
|  | >1000m | 54 (11.7%) | >1 | 173 (37.4%) | >10 | 184 (39.8%) |
| **Other specialized food stores ^a^** | | |  |  |  |  |
|  | <500m | 164 (35.5%) | 0 | 260 (56.3%) | 0-3 | 226 (48.9%) |
|  | 500-1000m | 149 (32.3%) | 1 | 89 (19.3%) | 4-10 | 139 (30.1%) |
|  | >1000m | 149 (32.3%) | >1 | 113 (24.5%) | >10 | 97 (21.0%) |
| **Small grocery stores** | |  |  |  |  |  |
|  | <500m | 198 (42.9%) | 0 | 230 (49.8%) | 0-3 | 161 (34.8%) |
|  | 500-1000m | 129 (27.9%) | 1 | 77 (16.7%) | 4-10 | 117 (25.3%) |
|  | >1000m | 135 (29.2%) | >1 | 155 (33.5%) | >10 | 184 (39.8%) |
|  |  |  |  |  |  |  |
|  |  |  | **Relative density ^e^** | | **Relative density** | |
| **Food stores selling fruits and vegetables** | |  |  |  |  |  |
|  |  |  | <0.4 | 223 (48.3%) | <0.4 | 59 (12.8%) |
|  |  |  | 0.4-0.5 | 110 (23.8%) | 0.4-0.5 | 255 (55.2%) |
|  |  |  | >0.6 | 129 (27.9%) | >0.6 | 148 (32.0%) |
