# Supplementary material for: Associations between retail food environment and the nutritional quality of food purchases in French households: The Mont’Panier cross-sectional study
Source: PLoS One. 2022 Apr 27;17(4):e0267639. doi: 10.1371/journal.pone.0267639 (PMC9045620; doi:10.1371/journal.pone.0267639)
Supplement: S3 Table — a CI = Confidence Interval. (DOCX) [file pone.0267639.s003.docx]

|  | **Beta** | **95% CI ^a^** | **p-value** |
| --- | --- | --- | --- |
| **Household structure** |  |  | **0.011** |
| One adult |  |  |  |
| One adult with at least one child | -0.10 | -0.51, 0.31 | 0.6 |
| Multiple adults | **0.33** | **0.11, 0.55** | **0.003** |
| Multiple adults with at least one child | 0.10 | -0.15, 0.34 | 0.4 |
| **Income per unit of consumption** |  |  | **<0.001** |
| < 1110 €/month |  |  |  |
| 1110-2000 €/month | **0.34** | **0.11, 0.56** | **0.004** |
| > 2000 €/month | **0.47** | **0.24, 0.70** | **<0.001** |
| Does not wish to respond | **0.74** | **0.33, 1.1** | **<0.001** |
| **Age of household head** |  |  | **<0.001** |
| < 30 years |  |  |  |
| 30-50 years | **0.39** | **0.14, 0.64** | **0.003** |
| > 50 years | **0.82** | **0.56, 1.1** | **<0.001** |
