# Supplementary material for: Associations between retail food environment and the nutritional quality of food purchases in French households: The Mont’Panier cross-sectional study
Source: PLoS One. 2022 Apr 27;17(4):e0267639. doi: 10.1371/journal.pone.0267639 (PMC9045620; doi:10.1371/journal.pone.0267639)
Supplement: S4 Table — a CI = Confidence Interval; the presence of supermarkets and other specialized food stores around the home was not included in this multivariate model because it had p-values >0.2 in bivariate analyses. (DOCX) [file pone.0267639.s004.docx]

|  | **Beta** | **95% CI** ^a^ | **p-value** |
| --- | --- | --- | --- |
| **Presence of markets** |  |  | **0.027** |
| No | — | — |  |
| Yes | **-0.21** | **-0.39, -0.02** | **0.027** |
| **Presence of greengrocers** |  |  | **0.012** |
| No | — | — |  |
| Yes | **0.26** | **0.06, 0.47** | **0.012** |
| **Presence of bakeries** |  |  | 0.6 |
| No | — | — |  |
| Yes | -0.05 | -0.26, 0.16 | 0.6 |
| **Presence of small grocery stores** |  |  | 0.3 |
| No | — | — |  |
| Yes | -0.11 | -0.31, 0.10 | 0.3 |
| **Income per unit of consumption** |  |  | 0.4 |
| < 1110 €/month | — | — |  |
| 1110-2000 €/month | 0.03 | -0.38, 0.44 | 0.9 |
| > 2000 €/month | 0.19 | -0.02, 0.41 | 0.078 |
| Does not wish to respond | 0.09 | -0.18, 0.36 | 0.5 |
| **Household structure** |  |  | 0.072 |
| One adult | — | — |  |
| One adult with at least one child | 0.22 | -0.01, 0.46 | 0.062 |
| Multiple adults | **0.30** | **0.05, 0.55** | **0.021** |
| Multiple adults with at least one child | **0.41** | **0.00, 0.82** | **0.049** |
| **Age of household head** |  |  | **<0.001** |
| < 30 years | — | — |  |
| 30-50 years | 0.18 | -0.12, 0.47 | 0.2 |
| > 50 years | **0.52** | **0.22, 0.82** | **<0.001** |
| **Away-from-home food consumption** | -0.01 | -0.02, 0.00 | 0.056 |
