# Supplementary material for: Associations between retail food environment and the nutritional quality of food purchases in French households: The Mont’Panier cross-sectional study
Source: PLoS One. 2022 Apr 27;17(4):e0267639. doi: 10.1371/journal.pone.0267639 (PMC9045620; doi:10.1371/journal.pone.0267639)
Supplement: S5 Table — a CI = Confidence Interval; the number of supermarkets, markets, bakeries, other specialized stores and small grocery stores in activity space was not included in this multivariate model because it had p-values >0.2 in bivariate analyses. The activity space includes areas around the home, around household members’ places of main activity, and commuting routes between those places. (DOCX) [file pone.0267639.s005.docx]

|  | **Beta** | **95% CI** ^a^ | **p-value** |
| --- | --- | --- | --- |
| **Number of greengrocers** |  |  | **<0.001** |
| 0 |  |  |  |
| 1 | **0.77** | **0.27, 1.3** | **0.003** |
| >1 | **0.73** | **0.36, 1.1** | **<0.001** |
| **Income per unit of consumption** |  |  | **<0.001** |
| < 1110 €/month |  |  |  |
| 1110-2000 €/month | **0.87** | **0.42, 1.3** | **<0.001** |
| > 2000 €/month | **0.79** | **0.28, 1.3** | **0.002** |
| Does not wish to respond | 0.53 | -0.20, 1.3 | 0.2 |
| **Household structure** |  |  | 0.4 |
| One adult |  |  |  |
| One adult with at least one child | 0.01 | -0.39, 0.42 | >0.9 |
| Multiple adults | 0.17 | -0.05, 0.38 | 0.12 |
| Multiple adults with at least one child | 0.14 | -0.13, 0.41 | 0.3 |
| **Age of household head** |  |  | **<0.001** |
| < 30 years |  |  |  |
| 30-50 years | 0.14 | -0.16, 0.43 | 0.4 |
| > 50 years | **0.47** | **0.18, 0.77** | **0.002** |
| **Away-from-home food consumption** | -0.01 | -0.02, 0.00 | **0.033** |
| **Number of greengrocers * Income per unit of consumption** |  |  | 0.052 |
| 1 * 1110-2000 €/month | **-0.74** | **-1.5, -0.02** | **0.044** |
| >1 * 1110-2000 €/month | **-0.88** | **-1.4, -0.35** | **0.001** |
| 1 * > 2000 €/month | -0.52 | -1.2, 0.20 | 0.2 |
| >1 * > 2000 €/month | **-0.69** | **-1.3, -0.12** | **0.018** |
| 1 * Does not wish to respond | -0.19 | -1.4, 1.0 | 0.8 |
| >1 * Does not wish to respond | -0.16 | -1.0, 0.73 | 0.7 |
