# Supplementary material for: Associations between retail food environment and the nutritional quality of food purchases in French households: The Mont’Panier cross-sectional study
Source: PLoS One. 2022 Apr 27;17(4):e0267639. doi: 10.1371/journal.pone.0267639 (PMC9045620; doi:10.1371/journal.pone.0267639)
Supplement: S6 Table — a CI = Confidence Interval; the presence of markets, bakeries, other specialized stores (butcher’s, fishmonger’s and dairy stores) and small grocery stores in activity space was not included in this multivariate model because it had p-values >0.2 in bivariate analyses. The activity space includes areas around the home, around household members’ places of main activity and commuting routes between those places. (DOCX) [file pone.0267639.s006.docx]

|  | **< 1110 € / month**  **(N=130)** | | | **1110-2000 € / month**  **(N=157)** | | | **> 2000 € / month**  **(N=147)** | | | **Does not wish to respond**  **(N=28)** | | |
| --- | --- | --- | --- | --- | --- | --- | --- | --- | --- | --- | --- | --- |
|  | **β** | **95% CI** ^a^ | **p** | **β** | **95% CI** ^a^ | **p** | **β** | **95% CI** ^a^ | **p** | **β** | **95% CI** ^a^ | **p** |
| **Presence of supermarkets** |  |  | 0.6 |  |  | 0.8 |  |  | 0.9 |  |  | 0.6 |
| No |  |  |  |  |  |  |  |  |  |  |  |  |
| Yes | -0.13 | -0.60, 0.35 | 0.6 | 0.06 | -0.32, 0.44 | 0.8 | 0.03 | -0.44, 0.50 | 0.9 | 0.17 | -0.46, 0.80 | 0.6 |
| **Presence of greengrocers** |  |  | **0.002** |  |  | 0.4 |  |  | 0.7 |  |  | **0.004** |
| No |  |  |  |  |  |  |  |  |  |  |  |  |
| Yes | **0.73** | **0.26, 1.2** | **0.003** | -0.17 | -0.53, 0.19 | 0.4 | 0.08 | -0.39, 0.54 | 0.7 | **0.93** | **0.24, 1.6** | **0.012** |
| **Household structure** |  |  | 0.11 |  |  | 0.5 |  |  | >0.9 |  |  | 0.5 |
| One adult |  |  |  |  |  |  |  |  |  |  |  |  |
| One adult with at least one child | -0.15 | -0.94, 0.63 | 0.7 | 0.07 | -0.51, 0.66 | 0.8 | -0.11 | -1.4, 1.2 | 0.9 | 0.47 | -1.2, 2.2 | 0.6 |
| Multiple adults | **0.50** | **0.03, 1.0** | **0.038** | 0.00 | -0.34, 0.34 | >0.9 | 0.07 | -0.33, 0.47 | 0.7 | -0.07 | -0.70, 0.56 | 0.8 |
| Multiple adults with at least one child | -0.10 | -0.74, 0.55 | 0.8 | 0.29 | -0.11, 0.69 | 0.2 | 0.03 | -0.42, 0.49 | 0.9 | 0.74 | -0.69, 2.2 | 0.3 |
| **Age of household head** |  |  | 0.071 |  |  | 0.6 |  |  | **<0.001** |  |  | **0.002** |
| < 30 years |  |  |  |  |  |  |  |  |  |  |  |  |
| 30-50 years | 0.48 | -0.09, 1.1 | 0.10 | -0.02 | -0.52, 0.48 | >0.9 | 0.01 | -0.72, 0.74 | >0.9 | **2.8** | **0.88, 4.6** | **0.007** |
| > 50 years | **0.58** | **0.07, 1.1** | **0.027** | 0.15 | -0.36, 0.66 | 0.6 | 0.69 | -0.05, 1.4 | 0.068 | **2.0** | **0.23, 3.8** | **0.029** |
| **Away-from-home food consumption** | 0.00 | -0.02, 0.03 | 0.8 | -0.02 | -0.04, 0.00 | 0.050 | -0.01 | -0.03, 0.01 | 0.3 | **-0.05** | **-0.09, -0.01** | **0.012** |
