# Supplementary material for: Associations between retail food environment and the nutritional quality of food purchases in French households: The Mont’Panier cross-sectional study
Source: PLoS One. 2022 Apr 27;17(4):e0267639. doi: 10.1371/journal.pone.0267639 (PMC9045620; doi:10.1371/journal.pone.0267639)
Supplement: S7 Table — a CI = Confidence Interval; the presence of markets, bakeries, other specialized stores (butcher’s, fishmonger’s and dairy stores) and small grocery stores in activity space was not included in this multivariate model because it had p-values >0.2 in bivariate analyses. The activity space includes areas around the home, around household members’ places of main activity and commuting routes between those places. (DOCX) [file pone.0267639.s007.docx]

|  | **β** | **95% CI** *^a^* | **p-value** |
| --- | --- | --- | --- |
| **Presence of supermarkets** |  |  | 0.6 |
| No |  |  |  |
| Yes | 0.05 | -0.18, 0.29 | 0.6 |
| **Presence of greengrocers** |  |  | **<0.001** |
| No |  |  |  |
| Yes | **0.71** | **0.34, 1.1** | **<0.001** |
| **Income per unit of consumption** |  |  | **0.001** |
| < 1110 €/month |  |  |  |
| 1110-2000 €/month | **0.86** | **0.41, 1.3** | **<0.001** |
| > 2000 €/month | **0.78** | **0.27, 1.3** | **0.003** |
| Does not wish to respond | 0.52 | -0.21, 1.3 | 0.2 |
| **Household structure** |  |  | 0.5 |
| One adult |  |  |  |
| One adult with at least one child | -0.01 | -0.41, 0.40 | >0.9 |
| Multiple adults | 0.16 | -0.05, 0.37 | 0.14 |
| Multiple adults with at least one child | 0.11 | -0.15, 0.38 | 0.4 |
| **Age of household head** |  |  | **<0.001** |
| < 30 years |  |  |  |
| 30-50 years | 0.16 | -0.13, 0.45 | 0.3 |
| > 50 years | **0.49** | **0.19, 0.78** | **0.001** |
| **Away-from-home food consumption** | -0.01 | -0.02, 0.00 | **0.028** |
| **Presence of greengrocers *  Income per unit of consumption** |  |  | **0.007** |
| Yes * 1110-2000 €/month | **-0.85** | **-1.4, -0.34** | **0.001** |
| Yes * > 2000 €/month | **-0.65** | **-1.2, -0.10** | **0.021** |
| Yes * Does not wish to respond | -0.16 | -1.0, 0.69 | 0.7 |
